# Relationship between factors of mental and physical health in medical students

**DOI:** 10.1192/j.eurpsy.2026.11850

**Published:** 2026-07-31

**Authors:** E. L. Nikolaev, S. A. Yusupov, N. D. Kodirov, J. N. Daminov

**Affiliations:** 1 Social and Clinical Psychology Department, I.N. Ulianov Chuvash State University, Cheboksary, Russian Federation; 2 Department of Pediatric Surgery No 1; 3 Department of Pharmacognosy and pharmaceutical technology; 4 Faculty of Pharmacy, Samarkand State Medical University, Samarkand, Uzbekistan

## Abstract

**Introduction:**

When analyzing a medical condition of students, researchers pay more attention to their physical (somatic) health. For medical students, the issues of mental health are important as well. How are the factors of mental and physical health interrelated in students of one of the medical universities of Central Asia?

**Objectives:**

Our objective was to determine the factors of mental and physical health and interrelation between them in medical university students.

**Methods:**

Our respondents were 70 different year students of one of the state medical universities of Uzbekistan. We used a physical and mental health inventory as a tool. We statistically processed the received data and subjected them to a correlational analysis with a view to finding out any significant interrelations at the significance level of p<.05.

**Results:**

We can describe the risk factors of the respondents’ mental health as follows: susceptibility to high or moderate stress (45.71%), smoking cigarettes (31.43%) and vapes (10.00%), acceptability of suicides among students (8.58%). The level of the respondents’ physical health is characterized by such deviations as excessive body mass or obesity (24.29%), chronic diseases that require treatment (20.00%), including a surgical interference (12.86%). Further, we present valid interrelations between the mental and physical factors. Thus, the level of the perceived stress is positively interrelated with the frequency of headaches (r=.33; p<.05), availability of chronic somatic illnesses (r=.28; p<.05) and the frequency of medications intake (r=.24; p<.05), whereas it is negatively interrelated with self-assessment of health (r=-.30; p<.05). Higher frequency of smoking in medical students is accompanied by a higher level of alcohol consumption (r=.48; p<.05) and a lower anti-suicide barrier (r=-.24; p<.05). On the contrary, a higher level of adaptive personality characteristics is interrelated with a higher level of physical health. Thus, the general health index in medical students is correlated with the intensity of hardiness (r=.45; p<.05) and resilience (r=.33; p<.05).

**Conclusions:**

The information about the interrelation between mental and physical health factors in medical students can lay the foundation for important decisions concerning the development and implementation of university and regional programs aimed at taking care of the students’ health and developing their resilience as of future health care workers.

**Disclosure of Interest:**

None Declared

